# The role of cell death in the physiological and pathological processes of skeletal muscle

**DOI:** 10.3389/fphys.2025.1717233

**Published:** 2025-11-17

**Authors:** Hongyi Xu, Zihui Gao, Xinlei Yao, Jiacheng Sun, Bingqian Chen

**Affiliations:** 1 Jiangsu Key Laboratory of Tissue Engineering and Neuroregeneration, Key Laboratory of Neuroregeneration of Ministry of Education, Co-Innovation Center of Neuroregeneration, Nantong University, Nantong, Jiangsu, China; 2 Department of Orthopedics, Changshu Hospital Affiliated to Soochow University, First People’s Hospital of Changshu City, Changshu, Jiangsu, China

**Keywords:** skeletal muscle, apoptosis, necrosis, autophagy, pyroptosis, ferroptosis, cuproptosis, sarcopenia

## Abstract

Skeletal muscle is the largest metabolic and motor organ in the human body. It facilitates daily movement and maintains posture through contraction. It also acts as a core tissue for energy metabolism by participating in glucose uptake, lipid oxidation, and thermogenesis. Thus, it plays a vital role in regulating systemic metabolic homeostasis. Under physiological conditions, skeletal muscle maintains a dynamic regulatory network to coordinate multiple cellular processes for tissue homeostasis. Apoptosis selectively removes damaged myonuclei and maintains myofiber structural integrity. Necroptosis prevents excessive inflammatory responses. Autophagy degrades abnormal proteins and organelles to ensure cytoplasmic quality control. Additionally, pyroptosis supports immune surveillance. In pathological states, abnormal activation of cell death programs occurs. These include apoptosis, necrosis, autophagy, pyroptosis, and ferroptosis. Such dysregulation can lead to myonuclear loss, myofiber atrophy, and fibrosis. While previous reviews have often focused on individual cell death pathways, this review provides a novel, integrated perspective by systematically outlining the roles and regulatory mechanisms of multiple death modalities in skeletal muscle. The interactions and balances among these pathways collectively determine muscle fate. We further discuss the implications of this network across various pathological contexts, such as muscular dystrophy, sarcopenia, and sepsis-induced atrophy. Finally, we identify promising therapeutic targets arising from this integrated view and discuss the challenges and future directions for translating these findings into clinical strategies. This review provides a comprehensive theoretical foundation for understanding the pathogenesis and treatment of skeletal muscle-related diseases.

## 1 Introduction

Skeletal muscle is a crucial tissue in the human body. It plays key roles in motor activity and the regulation of glucose and lipid metabolism homeostasis (Chen et al., 2023a; Zhang et al., 2023). The decline in its function is closely associated with frailty and mortality in the elderly. Sarcopenia refers to the loss of skeletal muscle mass and function. It acts as a significant catalyst for health deterioration in older adults. This condition not only impairs motor performance but also accelerates the development of other systemic health problems (Jiang et al., 2024). With the growing aging population, health and medical issues among the elderly impose a substantial economic burden on families and society.

Skeletal muscle consists of various cell types. Myocytes serve as the fundamental structural and functional units. Multiple mononuclear myoblasts fuse to form muscle fibers (Yamakawa et al., 2020). A crucial cell population in skeletal muscle is the muscle satellite (stem) cell group. These cells influence muscle growth, post-injury repair, and regeneration. They possess self-renewal capacity and multi-directional differentiation potential. Under normal conditions, muscle satellite cells remain quiescent. They reside beneath the basal lamina of muscle fibers (Mauro, 1961; Chang and Rudnicki, 2014). In some cases, satellite cells undergo asymmetric division to replenish daily muscle fiber loss. This process maintains the stem cell pool and supports muscle homeostasis and growth. After muscle injury, quiescent satellite cells are rapidly activated. They proliferate quickly and differentiate into myoblasts. These myoblasts then fuse with damaged fibers or with each other to form new muscle fibers. This promotes muscle repair and growth (Kim et al., 2015). The diverse cell types in skeletal muscle carry out specialized functions, and their coordinated interactions are essential for maintaining the tissue’s structural integrity and physiological function.

Skeletal muscle is a vital component of the motor system. Its normal physiological function depends on cellular homeostasis, which inherently involves both cell death and cell proliferation. Cell death mechanisms are complex and encompass multiple forms of cell death. Under various physiological and pathological influences, skeletal muscle cells experience different types of death processes. Among these, apoptosis is a programmed cell death pathway. It plays a key role in skeletal muscle development and remodeling (Dirks and Leeuwenburgh, 2005; Yang et al., 2020). For instance, during muscle injury repair, damaged and irreparable cells are eliminated via apoptosis. This clearance creates space for new cell growth and tissue regeneration (Yang et al., 2020). Meanwhile, autophagic cell death represents another significant form of cell death. It occurs through excessive degradation of damaged proteins and organelles within the cell. This process leads to energy exhaustion and functional failure, ultimately resulting in cell death (Yang et al., 2020; Leduc-Gaudet et al., 2023a). When skeletal muscle encounters stress conditions such as nutrient deprivation or hypoxia, the autophagy mechanism becomes activated. This activation promotes cell death (Franco-Romero and Sandri, 2021). Furthermore, other cell death modalities like necroptosis also contribute to skeletal muscle physiology and pathology in specific contexts (Zhou et al., 2020). These diverse cell death pathways interweave and act synergistically. Collectively, they shape the structure and function of skeletal muscle.

Therefore, cell death is a crucial component in skeletal muscle growth and regeneration, and various forms of cell death play distinct roles in this process. Understanding the relationship between cell death and skeletal muscle growth and regeneration is important. This knowledge is essential for in-depth research into the specific mechanisms of cell death in skeletal muscle. It also aids in comprehending the development of muscle atrophy and muscle diseases. Furthermore, it supports the development of effective therapeutic strategies and promotes the maintenance of skeletal muscle health. Ultimately, this holds promise for bringing new breakthroughs in the clinical treatment of skeletal muscle-related diseases.

While numerous reviews have addressed cell death in various tissues, a comprehensive and integrative analysis focusing specifically on multiple regulated cell death pathways (including apoptosis, necroptosis, pyroptosis, ferroptosis, and autophagy) within the context of skeletal muscle physiology and pathology remains limited. The primary aim of this review is to systematically synthesize current knowledge on these diverse cell death modalities in skeletal muscle, explicitly contrasting their roles in maintaining tissue homeostasis under physiological conditions with their dysregulation in pathological states such as aging, disuse atrophy, and hereditary myopathies. A key novelty of our work lies in the proposal of an integrated conceptual framework that illustrates the balance among these pathways, highlighting how their imbalance contributes to muscle atrophy, impaired regeneration, and fibrosis. By delineating these complex interactions, we aim to identify critical gaps in the current understanding and underscore the translational potential of targeting specific cell death pathways for treating skeletal muscle disorders.

## 2 Literature search methodology

The literature for this narrative review was identified through systematic searches in major scientific databases, including PubMed and Web of Science. The search strategy utilized a combination of keywords and Medical Subject Headings (MeSH) terms related to “skeletal muscle,” “muscle atrophy,” “sarcopenia,” and specific cell death modalities such as “apoptosis,” “necroptosis,” “pyroptosis,” “ferroptosis,” “cuproptosis,” and “autophagy.” The search was focused on articles published up to [2025] to ensure the inclusion of the most recent findings. The initial search results were screened based on their titles and abstracts. Full-text articles were then assessed for eligibility. The primary inclusion criteria were (Chen et al., 2023a): original research articles or high-impact reviews (Zhang et al., 2023); studies directly investigating the role of cell death pathways in skeletal muscle physiology or pathology; and (Jiang et al., 2024) publications in English.

## 3 Basic structure and function of skeletal muscle

The skeletal muscle surface is enveloped by dense connective tissue known as the epimysium. This layer separates the muscle from surrounding tissues. In cross-section, the peripheral epimysium and internal muscle bundles are visible. Each bundle is surrounded by connective tissue called the perimysium. The perimysium contains blood vessels, nerves, and lymphatic vessels. Muscle bundles consist of multiple muscle fibers. A thin connective tissue layer called the endomysium covers each muscle fiber. It contains capillaries, neuronal axons, and muscle satellite cells. Muscle satellite cells regulate skeletal muscle growth, repair, and regeneration (Frontera and Ochala, 2015). In a quiescent state, these cells exhibit low transcriptional activity and remain in the G0 phase. External stimuli can prompt them to enter the cell cycle. They then differentiate into new muscle fibers for repair or to replenish the satellite cell pool (Yamakawa et al., 2020). However, aging or homeostatic disruption in muscle tissue primarily impairs satellite cell regenerative capacity (Hikida, 2011). This results from intrinsic cellular changes or alterations in the muscle microenvironment (Dumont et al., 2015; Blau et al., 2015). Skeletal muscle fibers are multinucleated cells with hundreds of nuclei. Muscle size is mainly determined by individual fiber size and fiber number (Argilés et al., 2016). Other studies indicate that myogenic interstitial progenitor cells (IPCs) reside in skeletal muscle interstitial regions. These cells are multipotent and include fibro/adipogenic progenitors (FAPs) or mesenchymal stromal cells. IPCs secrete various cytokines, growth factors, and extracellular matrix components. They regulate the quiescence and activation of muscle stem cells and maintain tissue homeostasis (Uezumi et al., 2010). Upon skeletal muscle injury, IPCs become activated. They proliferate and differentiate into myoblasts, contributing to muscle repair and regeneration (Jiang et al., 2024; Sastourné-Arrey et al., 2023). Thus, a deeper understanding of satellite cell and IPC interactions will provide crucial insights for treating muscle-related diseases and advancing regenerative medicine.

Skeletal muscle interacts with various organs and this crosstalk weaves the entire life into a vast biological network. It primarily involves motor function, energy metabolism, and substance metabolism (Yi et al., 2025). First, skeletal muscle is essential for maintaining body posture and motor function. At the molecular level, myosin and actin cooperate perfectly. ATP hydrolysis provides energy for sarcomere shortening and tension generation (Frontera and Ochala, 2015). At the organ level, skeletal muscle supplies pulling force and works with the skeletal and nervous systems. This collaboration enables complex human movements (Severinsen and Pedersen, 2020). Second, skeletal muscle is the body’s largest glucose storage organ. Its fiber types define the energy metabolism model. Type I fibers are rich in mitochondria and sustain low-intensity contractions through fatty acid β-oxidation. Type II fibers depend on glycolysis for rapid energy production and support explosive movements (Qaisar et al., 2016; Schiaffino and Reggiani, 2011). Exercise activates AMPK and PGC-1α. This promotes mitochondrial biogenesis and enhances insulin sensitivity (Eisele and Handschin, 2014; Chen et al., 2023b). Recent studies show that skeletal muscle also has endocrine functions. It secretes myokines which regulate lipolysis and bone metabolism via paracrine and autocrine mechanisms (Pedersen and Febbraio, 2008). For instance, muscle-derived insulin-like growth factor 1 (IGF-1) acts on osteoblasts expressing IGF-1 receptors. This action stimulates bone formation (Yi et al., 2025; Perrini et al., 2010). In summary, skeletal muscle possesses a unique structure and diverse functions. It plays a vital role in human movement, posture maintenance, and protection.

## 4 Major types and characteristics of cell death

Cell death is a critical physiological and pathological process, intricately linked to development, tissue homeostasis, and the pathogenesis of numerous diseases. The most common form is apoptosis, which is characterized by cell shrinkage, membrane blebbing, apoptotic body formation, DNA fragmentation, and chromatin condensation (Kerr, 1971). Apoptosis plays important roles in development, tissue remodeling, and disease processes. Research has identified two main apoptotic pathways, the extrinsic pathway and the intrinsic pathway. The extrinsic apoptotic pathway is also called the death receptor pathway and it is mediated by external signals interacting with transmembrane receptors. This pathway involves death receptors from the tumor necrosis factor (TNF) receptor gene superfamily (Locksley et al., 2001). TNF receptor family members share a similar domain known as the death domain, an approximately 80-amino acid cytoplasmic region that transmits death signals from the cell surface to the intracellular space (Ashkenazi and Dixit, 1998). Ligands and their corresponding death receptors include Fas ligand (FasL) and Fas receptor (FasR), TNF-α/Tumor necrosis factor receptor 1 (TNFR1), Apo3L/DR3, Apo2L/DR4, and Apo2L/DR5 (Ashkenazi and Dixit, 1998; Chicheportiche et al., 1997; Peter and Krammer, 1998; Rubio-Moscardo et al., 2005). Death receptor-ligand binding recruits various adaptor proteins, for example, Fas ligand-receptor binding recruits Fas-associated protein with death domain (FADD) while TNF ligand-receptor binding recruits TNFR1-associated death domain protein (TRADD) along with FADD and receptor-interacting protein (RIP) (Hsu et al., 1995; Kelliher et al., 1998). FADD binds to caspase-8 through death domain dimerization, forming the death-inducing signaling complex (DISC) and leading to self-catalytic activation of caspase-8. Once caspase-8 is activated, it triggers the execution phase of apoptosis. Activated caspase-8 then activates downstream effector caspases, ultimately resulting in programmed cell death. This completes the core steps of the extrinsic apoptotic pathway and prepares for the subsequent execution phase and the intrinsic pathway.

The endogenous apoptosis pathway is the intrinsic signaling pathway of apoptosis. It directly acts on intracellular signals and is also called the mitochondrial pathway. When intracellular homeostasis is disrupted, changes occur in the mitochondrial inner membrane. The mitochondrial permeability transition pore (MPTP) opens and the mitochondrial transmembrane potential is lost. This leads to the release of pro-apoptotic factors such as cytochrome c. These factors activate the caspase-dependent mitochondrial pathway. Cytochrome c binds to apoptotic protease-activating factor 1 (Apaf-1) and recruits caspase-9 to form the apoptosome. This activates downstream caspase-3 and caspase-7, resulting in DNA fragmentation and cell disintegration (Saelens et al., 2004; Du et al., 2000; Chinnaiyan, 1999; Hill et al., 2004). Furthermore, other pro-apoptotic factors including apoptosis-inducing factor (AIF), endonuclease G, and caspase-activated DNase (CAD) are released from mitochondria. AIF translocates to the nucleus and causes DNA fragmentation along with peripheral nuclear chromatin condensation (Joza et al., 2001). Endonuclease G also moves to the nucleus and cleaves chromatin to generate oligonucleosomal DNA fragments (Li et al., 2001). Subsequently, CAD translocates to the nucleus and is cleaved by caspase-3. This causes oligonucleosomal DNA fragmentation and more severe chromatin condensation (Enari et al., 1998). Therefore, the endogenous apoptosis pathway precisely and orderly executes programmed cell death through multiple mitochondria-mediated molecular events.

The regulation of mitochondrial events in apoptosis is mediated by the B-cell lymphoma 2 (Bcl-2) protein family (Cory and Adams, 2002). This family controls the release of cytochrome c by modulating mitochondrial membrane permeability. The tumor suppressor p53 plays a key role in regulating the Bcl-2 protein family (Greenhalgh, 1998). During the final execution phase of apoptosis, initiator caspases such as caspase-8, -9, and -10 work together with effector caspases including caspase-3, -6, and -7. Among these, caspase-3 is considered the primary executioner caspase. It activates the endonuclease CAD, leading to chromosomal DNA degradation and chromatin condensation (Elmore, 2007). In skeletal muscle, excessive mechanical stress, nutritional deficiencies, or certain pathological conditions can induce apoptosis in myocytes. This process subsequently affects muscle mass and function (Kjaer, 2004; Landi et al., 2019). Studies have shown significantly elevated levels of apoptosis in the skeletal muscle of patients with muscular atrophy. This may contribute to muscle fiber shrinkage and loss of muscle strength (Dirks and Leeuwenburgh, 2005; Yang et al., 2020). Therefore, a deeper understanding of the molecular mechanisms of skeletal muscle apoptosis is crucial for developing new therapeutic strategies for muscle-wasting diseases.

Necrosis is another important form of cell death. It results from pathological damage to local tissue cells and is usually caused by external factors such as hypoxia, toxins, and infections (Vanden et al., 2014). Its characteristics include cell swelling, organelle membrane rupture, lysosomal swelling and rupture, and ultimately the destruction of cell membrane integrity (Weinlich et al., 2017). This leads to the release of cellular contents into the tissue, which triggers an inflammatory response and recruits macrophages or adjacent cells for phagocytosis (Savill and Fadok, 2000). Histologically, apoptosis can be difficult to distinguish from necroptosis, and the two processes may occur simultaneously. However, necroptosis is an uncontrolled passive process that typically affects a large area of cells, while apoptosis is a regulated and controlled process affecting only individual cells or small groups. Necrotic cell death has long been considered unregulated (Zeiss, 2003). Necroptosis has two core components, receptor-interacting protein kinase 3 (RIPK3) and mixed lineage kinase domain-Like protein (MLKL) (Zhang J. et al., 2024). It can be triggered by various mechanisms, including the activation of death receptors such as FAS and TNFRSF1A, toll-like receptors like TLR3 and TLR4, and TNFα (Holler et al., 2000; He et al., 2011). In the activation of TNFα signaling, TNFα binds to its receptor TNFR1 and recruits other proteins to form complex I. This complex includes TRADD, FADD, receptor-interacting protein kinase 1 (RIPK1), TNFR-associated factors (TRAF), and cellular inhibitors of apoptosis 1 (clAP1) and clAP2. FADD recruits procaspase-8, and RIPK1 recruits RIPK3 (Cho et al., 2009). This represents a critical turning point in cell death. If caspase-8 is activated, it leads to apoptosis. However, when caspase-8 activity is inhibited, the RIPK3 and RIPK1 complex recruits and phosphorylates MLKL (Cho et al., 2009; Sun et al., 2012). This forms the necrosome complex, which promotes necroptosis. During the necroptosis stage, MLKL phosphorylation causes conformational changes and insertion into the cell membrane or organelle membranes (Dondelinger et al., 2014). This results in ion imbalance and osmotic swelling, ultimately leading to cell membrane rupture and the release of inflammatory factors (Zhao et al., 2012; Wang et al., 2014). Therefore, in-depth research on the mechanisms of necroptosis not only aids in understanding the pathological processes of related diseases but also offers new perspectives for developing therapeutic strategies against inflammatory and degenerative diseases.

In recent years a new form of cell death called ferroptosis has been discovered. Ferroptosis is driven by the iron-dependent accumulation of lipid reactive oxygen species (ROS), ultimately leading to cell death (Liu et al., 2022). Its characteristic feature is a reduction in mitochondrial volume. Cells depend on two major antioxidant systems the GSH system and the CoQ10 system to clear ROS and maintain redox stability (Mugoni et al., 2013; Jazvinšćak Jembrek et al., 2014; Schafer and Buettner, 2001). Glutathione consists of glutamate cysteine and glycine (Forman et al., 2009). Glutathione peroxidase 4 (GPX4) is a selenoprotein with selenocysteine at its active center (Ingold et al., 2018). GPX4 converts toxic lipid hydroperoxides into non-toxic lipid alcohols (L-OH). This action prevents Fe^2+^-dependent lipid ROS accumulation, thereby inhibiting ferroptosis (Dang et al., 2022). The system xc^−^ comprises the regulatory subunit solute carrier family 3 member 2 (SLC3A2) and the catalytic subunit solute carrier family 7 member 11 (SLC7A11) (Bridges et al., 2012). This complex mediates the exchange of extracellular cystine and intracellular glutamate across the plasma membrane. Intracellular cystine is then reduced to cysteine which is necessary for glutathione (GSH) production (Bridges et al., 2012). Coenzyme Q10 (CoQ10) is a potent lipid-soluble antioxidant (Laredj et al., 2014). It recruits ferroptosis suppressor protein 1 (FSP1) to the plasma membrane. FSP1 reduces CoQ10 using NAD(P)H and this process suppresses lipid peroxidation (Bersuker et al., 2019). Studies on skeletal muscle aging have revealed increased iron ion accumulation in muscle tissue. This may trigger ferroptosis leading to impaired muscle function and atrophy (Wang Y. et al., 2022). Changes in ferroptosis-related indicators have also been observed suggesting ferroptosis contributes to the development and progression of musculoskeletal diseases. Therefore, exploring the specific mechanisms of ferroptosis in muscle aging and related disorders may provide new targets for future therapeutic interventions.

Pyroptosis is a form of regulated cell death (RCD) activated by inflammasomes that ultimately results in the loss of plasma membrane integrity. Inflammasomes are cytosolic multiprotein complexes which release interleukin-1 family cytokines, bind the adaptor molecule ASC (apoptosis-associated speck-like protein containing a CARD), and activate pro-inflammatory caspase-1 (Tang et al., 2019). Two triggering models exist for inflammasomes. Under conditions such as infection, tissue damage, or metabolic imbalance, both canonical (caspase-1) and non-canonical (caspase-4/5/11) inflammasomes can be activated (Broz and Dixit, 2016). Pathogen-associated molecular patterns (PAMPs) or damage-associated molecular patterns (DAMPs) can also activate inflammasomes through pattern recognition receptors. Research indicates that Gasdermin D (GSDMD) is a key downstream protein in the pyroptosis mechanism (Chen et al., 2016; Sborgi et al., 2016). GSDMD is cleaved by caspase-1 or caspase-11, releasing its N-terminal domain (GSDMD-N) (Kayagaki et al., 2015). This domain translocates to the plasma membrane and induces membrane permeabilization, leading to osmotic imbalance, cell swelling, and eventual rupture (Kayagaki et al., 2015; Ding et al., 2016). Unlike apoptosis, pyroptosis does not involve DNA fragmentation *in vitro* (Chen et al., 2016). Furthermore, in the classical caspase-1-dependent pathway, the precursors of IL-1β and IL-18 are cleaved to generate mature inflammatory cytokines, which are released extracellularly through pores (Tang et al., 2019; Rathkey et al., 2018). Thus, pyroptosis is not only a form of cell death but also an essential part of the immune response.

Autophagy is a lysosome-dependent degradation pathway that recycles intracellular components and removes damaged organelles. It is generally viewed as a pro-survival process activated by stress stimuli (Zhang et al., 2023; Bonaldo and Sandri, 2013). However, accumulating evidence indicates that autophagy can also drive cell death (Huang et al., 2025). In mammals, three distinct mechanisms deliver autophagic cargo to lysosomes. These include macroautophagy, chaperone-mediated autophagy, and microautophagy (Bonaldo and Sandri, 2013; Wang W. et al., 2022). In muscle, most autophagic processes involve macroautophagy. Macroautophagy initiation depends on the AMPK and mTOR pathways. AMPK reduces the inhibitory effect of mTOR on ULK1 complex formation (Green, 2019), which promotes autophagic vesicle generation (Brier et al., 2019). The Beclin-1/VPS34 complex then supports vesicle elongation. ATG family proteins subsequently facilitate fusion with autophagosomes (Zhou et al., 2022). LC3-II is recruited into autophagosomes with help from ATG3 and ATG7. Finally, the SNARE complex mediates autophagosome-lysosome fusion to form autolysosomes (Shen et al., 2021). Thus, precise regulation of autophagy is essential for cellular homeostasis and muscle function.

## 5 The role of cell death in skeletal muscle

**FIGURE 1:**
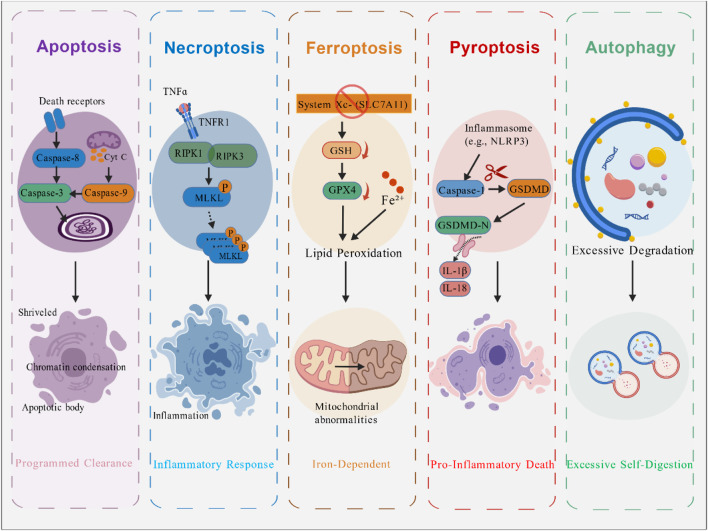
Major Types of Cell Death in Skeletal Muscle. This figure compares the molecular mechanisms and hallmark features of the major regulated cell death pathways implicated in skeletal muscle. These include: Apoptosis, characterized by caspase activation, DNA fragmentation, and formation of apoptotic bodies; Necroptosis, a programmed necrosis mediated by receptor-interacting protein kinase 1 (RIPK1)/RIPK3/mixed lineage kinase domain-like protein (MLKL) activation leading to membrane rupture and inflammation; Ferroptosis, an iron-dependent form driven by lipid peroxidation and glutathione peroxidase 4 (GPX4) inhibition; Pyroptosis, an inflammatory death initiated by inflammasome activation, Caspase-1/gasdermin D (GSDMD)-mediated pore formation, and release of interleukin-1β (IL-1β)/IL-18; and Autophagy, associated with excessive autophagosome formation and lysosomal degradation. The diagram underscores their distinct triggers, executors, and morphological outcomes. Figure created by BioGDP.

Striated muscle is the largest tissue in the human body accounting for about 40% of total body mass and serves as the primary amino acid reservoir. Cell death plays a dual role in skeletal muscle being essential for normal development and homeostasis maintenance while also acting as a key factor in various pathological conditions. It is of great significance to clarify the molecular mechanisms and typical characteristics of the main cell death pathways that are regulated in skeletal muscles (Figure 1; Table 1). By regulating cell death processes, it is possible to potentially improve muscle function delay muscle atrophy and promote repair and regeneration after injury.

**TABLE 1:** Cell death pathways in skeletal muscle.

Cell death	Key regulators	Physiological roles	Pathological roles and associated diseases
Apoptosis	FasL/FasR, TNF-α/TNFR1, FADD, cytochrome c, Caspase-3, Caspase-8, Caspase-9, Bcl-2 family	• Sculpting muscle during embryogenesis • Maintaining myonuclear domain by removing damaged/senescent nuclei • Regulating satellite cell pool homeostasis	Disuse atrophy: mitochondrial apoptotic pathway Denervation of skeletal muscle: activating caspase-9 and caspase-3 Aged muscle: non-caspase-dependent pathway
Necroptosis	RIPK1, RIPK3, MLKL, TNF-α, FAS, TLR3/4	Potential pro-regenerative role via DAMP release (e.g., Tenascin-C) to activate muscle stem cells	• Duchenne Muscular Dystrophy: Primary mode of myofiber death; RIPK1/RIPK3/MLKL upregulated • Inflammatory Myopathy: Synergistic with pyroptosis • Aging: Contributes to sterile inflammation
Ferroptosis	Iron accumulation, lipid peroxidation, GPX4, System xc^−^, FSP1/CoQ10, p53/SLC7A11 axis	Normal iron turnover	• Sarcopenia: Iron accumulation impairs satellite cell function • Osteoarthritis: Senescent macrophages induce muscle ferroptosis • Muscle contusion: Iron overload activates SAT1/ALOX15
Pyroptosis	NLRP3, caspase-1/4/5/11, GSDMD, IL-1β, IL-18	Immune surveillance; anti-tumor immunity	• Sepsis-Induced Atrophy: Core pathway via NLRP3/GSDMD; upregulates MuRF1/MAFbx • Muscle atrophy: Dexamethasone-induced • Type II Diabetes: Mitochondrial ROS activates NLRP3
Autophagy	AMPK/mTOR, ULK1 complex, beclin-1/VPS34, ATG proteins, LC3, PINK1/Parkin, Bnip3	• Clearance of damaged proteins/organelles, quality control • Exercise-induced metabolic enhancement • Selective mitophagy for mitochondrial homeostasis	• Aging Muscle: Declining autophagic flux, organelle accumulation • Denervation/Fasting: Disrupted mitochondrial network dynamics • Congenital Dystrophy: Impaired mitophagy

### 5.1 The role of apoptosis in skeletal muscle

Apoptosis plays a dual role in skeletal muscle, exerting vital physiological functions during development and homeostasis, while its dysregulation contributes significantly to various pathological conditions.

During embryonic development, apoptosis is essential for sculpting the nascent muscle. It fine-tunes myofiber size and number by eliminating excess myoblasts that fail to integrate properly into forming myotubes, ensuring the proper architecture of the muscle tissue (Sambasivan and Tajbakhsh, 2007; Parker et al., 2003). This process is crucial for the removal of misplaced or developmentally impaired cells, thereby refining the muscle pattern (Yong et al., 2020; Cao et al., 2021). In adult skeletal muscle, a basal level of apoptosis persists as a homeostatic mechanism. It contributes to the maintenance of the myonuclear domain by selectively removing individual, damaged, or senescent myonuclei without compromising the entire syncytial fiber (Zhang Y. et al., 2024). This selective culling is vital for preserving the functional integrity and quality of the muscle fibers, facilitating a slow but constant cellular turnover. Furthermore, apoptosis is involved in regulating the satellite cell pool, helping to maintain the balance between self-renewal and differentiation (Wu et al., 2023), which is critical for the long-term regenerative capacity of the muscle.

In contrast to its beneficial physiological roles, the abnormal activation of apoptosis is a well-established key mechanism driving skeletal muscle atrophy across diverse pathologies. When the apoptotic response becomes dysregulated during aging, damaged cells accumulate in mitotic tissues, which may lead to higher cancer incidence or functional decline (Warner, 1999). In skeletal muscle atrophy, excessive activation of apoptosis is a key mechanism. It has been confirmed that myocyte apoptosis occurs in muscular dystrophy, disuse atrophy, denervation of skeletal muscle, diabetic muscle atrophy, and exercise-induced muscle injury (Yang et al., 2020). In denervation models, mitochondrial dysfunction in muscle releases cytochrome C, activating caspase-9 and caspase-3, which triggers apoptosis and results in muscle dysfunction (Yang et al., 2020). Prolonged bed rest or microgravity in space leads to disuse atrophy of skeletal muscle. Studies in rat hindlimb suspension models show that mitochondrial dysfunction in muscle fibers causes reactive oxygen species (ROS) accumulation (Ji et al., 2024), activating the mitochondrial apoptotic pathway and subsequently caspase-3, thereby inducing myocyte apoptosis (Leeuwenburgh et al., 2005). Research indicates that muscle mass and fiber number decrease significantly with aging. A key molecular mechanism linking mitochondrial dysfunction to apoptosis in aging muscle involves the downregulation of Mitochondrial Calcium Uptake Family Member 3 (MICU3). This impairment disrupts mitochondrial calcium homeostasis, leading to increased oxidative stress and apoptosis, thereby contributing to sarcopenia; conversely, restoring MICU3 can mitigate these effects (Yu et al., 2025). In aged animals, the mechanisms of apoptosis exhibit distinct features. Apoptosis levels are higher in aged muscle, as confirmed by TUNEL staining and DNA fragmentation assays, but the molecular pathways involved tend to favor non-caspase-dependent mechanisms (Leeuwenburgh et al., 2005). For instance, mitochondrial release of apoptosis-inducing factor (AIF) and endonuclease G (EndoG) leads to their nuclear translocation (Tews et al., 1997). In aged mice subjected to hindlimb suspension, EndoG protein localization increases in myonuclei, while caspase-3 activity shows no significant change (Dupont-Versteegden, 2005). In the context of sarcopenia, this shift towards caspase-independent apoptosis represents a key mechanistic feature of aging muscle, contributing to the gradual loss of myonuclei and muscle fibers. Notably, in sepsis, dysregulated apoptosis is a major contributor to muscle wasting. One mechanism involves the long non-coding RNA MALAT1, which recruits EZH2 to suppress BRCA1 expression, thereby promoting skeletal muscle cell apoptosis and inflammatory responses (Yong et al., 2020). Simultaneously, sepsis has been shown to inhibit myoblast proliferation and promote apoptosis through the downregulation of the PLK1-AKT signaling pathway, further accelerating muscle atrophy (Cao et al., 2021). These findings illustrate distinct molecular pathways through which sepsis triggers apoptotic cell death and compromises muscle regeneration, collectively driving the rapid decline in muscle mass.

In summary, apoptosis plays a context-dependent role in skeletal muscle. Physiological apoptosis is indispensable for developmental morphogenesis and adult tissue homeostasis, whereas pathological apoptosis directly causes myonuclear loss and muscle fiber atrophy, contributing to functional decline.

### 5.2 The role of necroptosis in skeletal muscle

Necroptosis represents a regulated form of necrosis that plays context-dependent roles in skeletal muscle, contributing to both homeostatic regeneration and pathological damage.

Emerging evidence suggests that necroptosis is not merely a pathological cell death pathway but may also be co-opted into physiological processes that support tissue homeostasis and repair. The skeletal muscle is an immunologically unique tissue where the timely clearance of dead cells and the resolution of inflammation are critical for effective regeneration (Chen et al., 2023a). In this context, a carefully controlled level of necroptotic signaling may contribute to the creation of a regenerative microenvironment. Studies indicate that the execution of necroptosis in a subset of myofibers can function as a pro-regenerative signal. Specifically, the increased expression of necroptosis components and phosphorylation of MLKL in myofibers lead to the release of damage-associated molecular patterns (DAMPs) and specific matricellular proteins, such as Tenascin-C (TNC) (Zhou et al., 2020). TNC, in turn, promotes muscle stem cell (MuSC) proliferation and activation, thereby facilitating robust muscle regeneration (Zhou et al., 2020). This process underscores the dual nature of inflammatory signaling, where a typically destructive pathway can be harnessed to initiate and coordinate a reparative response, ensuring the restoration of tissue integrity after injury.

**FIGURE 2:**
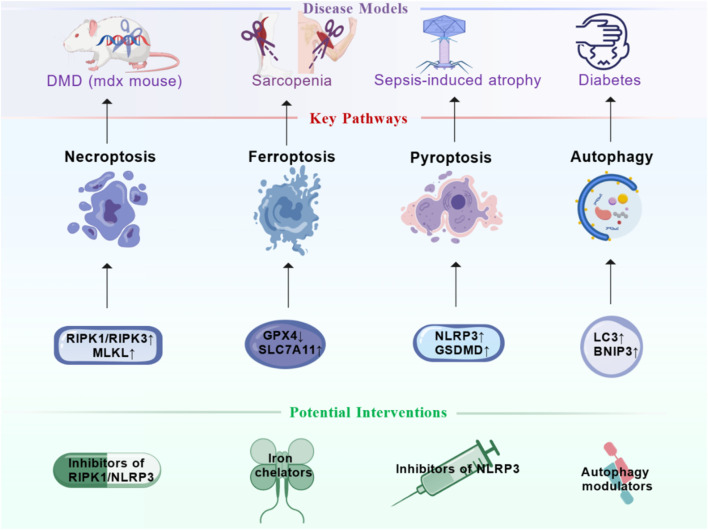
Therapeutic Targeting of Cell Death Pathways in Skeletal Muscle Disorders. This figure summarizes promising therapeutic strategies for skeletal muscle disorders by targeting specific cell death pathways. It links established disease models (e.g., duchenne muscular dystrophy (DMD)/mdx mouse, sarcopenia, sepsis-induced atrophy, diabetes) to their prominently dysregulated death pathways—necroptosis (RIPK1/MLKL), ferroptosis (GPX4/solute carrier family 7 member 11 (SLC7A11)), pyroptosis (nucleotide-binding oligomerization domain-like receptor protein 3 (NLRP3)/GSDMD), and autophagy (microtubule-associated protein 1 light chain 3 (LC3)/BCL2 Interacting Protein 3 (BNIP3)). Corresponding potential interventions are proposed, including caspase inhibitors, RIPK1 inhibitors, NLRP3 inflammasome inhibitors, iron chelators, and autophagy modulators. Figure created by BioGDP.

In contrast to its potential physiological roles, the dysregulated activation of necroptosis is a well-established driver of pathology in various skeletal muscle disorders. When myocardial infarction affects skeletal muscle blood supply, skeletal muscle cells undergo necrosis due to hypoxia. This manifests as muscle pain and weakness among other symptoms (Joyner and Casey, 2015; Richardson et al., 2000). Studies have detected nuclei with apoptotic DNA fragmentation in muscles of DMD patients and in the DMD mouse model mdx (Matsuda et al., 1995). However, this is relatively rare, indicating that apoptotic death contributes minimally to myofiber loss. In contrast, necrotic morphology characterizes most degenerating myofibers in DMD (Carpenter and Karpati, 1979; Sand et al., 1999). It is well known that DMD results from the absence of the dystrophin gene. Lack of dystrophin renders myofibers more susceptible to damage (Rosenberg et al., 2015). Inflammatory processes release cytokines involved in muscle injury, and tumor necrosis factor-α (TNFα) exerts a strong pro-necrotic effect in mdx myofibers (Grounds and Torrisi, 2004; Hodgetts et al., 2006; Chang et al., 2023). Furthermore, research shows that necroptosis-related proteins RIPK1, RIPK3, and MLKL are significantly upregulated in mdx mouse muscle (Morgan et al., 2018a). Therefore, inhibiting RIPK1/RIPK3 may become a potential intervention for improving the condition of DMD (Figure 2). Recent research has significantly expanded our understanding of the regulatory networks governing necroptosis and its crosstalk with other cell death pathways in skeletal muscle pathology. In idiopathic inflammatory myopathy, for instance, deficiency of muscle-derived brain-derived neurotrophic factor (BDNF) enhances mitochondrial reactive oxygen species (mtROS) accumulation, which sensitizes cells to TNFα-induced RIPK activation and promotes both necroptosis and pyroptosis. This demonstrates a synergistic interplay between different regulated cell death forms in driving muscle inflammation (Leduc-Gaudet et al., 2023b). This crosstalk is further exemplified by the concept of PANoptosis—a coordinated cell death process involving apoptosis, necroptosis, and pyroptosis. In a rat model of post-stroke sarcopenia, the extract of Chrysanthemum Flos was found to mitigate muscle atrophy by simultaneously inhibiting key mediators of all three PANoptosis pathways, including ZBP1, GSDMD, cleaved caspase-3/6/8, and p-MLKL/p-RIPK1/p-RIPK3 (Vazeille et al., 2012). The role of necroptosis in age-related muscle decline is supported by evidence showing that MLKL-deficient female mice are protected against age-related sterile inflammation and exhibit fewer immune cell infiltrates and regenerating myocytes in connective tissue and skeletal muscle, implicating MLKL in the chronic inflammatory milieu of aging muscle (Wang et al., 2018). These findings collectively highlight necroptosis as a dynamically regulated process influenced by metabolic factors, oxidative stress, and intercellular signaling, extending its role beyond a mere executor of cell death to a modifiable target in complex muscle disorders.

### 5.3 The role of ferroptosis in skeletal muscle

In recent years, the role of ferritin deficiency in various skeletal muscle diseases has drawn researchers’ attention and it has been reported as a key player in physiological and pathological processes (Lewerenz et al., 2013). Sarcopenia involves the degeneration or loss of muscle mass and strength due to an imbalance between muscle synthesis and degradation (Tan et al., 2020). Studies have found that with aging, the number or function of muscle satellite cells declines, which severely impairs skeletal muscle self-renewal and regeneration capacity (Budai et al., 2018). It has been demonstrated that unstable iron accumulation occurs in aged skeletal muscle and has been shown to contribute to muscle damage in mice by downregulating paired box protein 7 (Pax7) and myogenic factor 5 and inhibiting C2C12 myoblast differentiation (Ikeda et al., 2019). TFR1 is an important factor for muscle satellite cell activation and proliferation, and its expression is significantly reduced in aged skeletal muscle. Research indicates that deletion of the TFR1 gene promotes non-heme iron uptake by the zinc transporter Zip14 (Slc39a14), downregulates GPX4, Nrf2, and FTH1, accelerates iron accumulation and lipid peroxidation levels, thereby activating ferroptosis in skeletal muscle cells and impairing regeneration (Ding et al., 2021). Furthermore, C2C12 myoblasts also exhibit age-related iron accumulation, and activation of the p53/SLC7A11 axis induces ferroptosis in C2C12 cells, impairing their differentiation from myoblasts to myotubes (Huang et al., 2021). Beyond iron overload itself, transcriptional regulation of ferroptosis in muscle stem cells is also critical. Research by Maimaiti et al. revealed that in aged muscle, the transcription factor FOXO1 upregulates TXNIP, which in turn suppresses glutathione metabolism, thereby inducing ferroptosis in satellite cells and exacerbating the decline of muscle regenerative capacity (Maimaiti et al., 2025). Thus, ferroptosis emerges as an important mechanism in aging skeletal muscle, driven by both metabolic disruption and transcriptional reprogramming.

Emerging evidence further links ferroptosis to a broader spectrum of muscle pathologies, underscoring its role as a central cell death mechanism. In osteoarthritis (OA), for example, senescent macrophages in the quadriceps muscle promote atrophy by paracrine induction of ferroptosis; this process involves iron overload-induced mitochondrial damage, which reduces asparagine metabolites and impairs CoQ10 synthesis via suppressed mTORC1-HMGCR signaling, ultimately enhancing lipid peroxidation (Wang D. et al., 2022). Similarly, in chronic kidney disease (CKD)-related muscle injury, the natural compound Lobetyolin alleviates ferroptosis and muscle wasting by activating the Hedgehog-GLI1 signaling pathway, upregulating key ferroptosis suppressors (Tabata et al., 2019). Recent work also identified Nestin as a critical regulator of autophagy-dependent ferroptosis in muscle atrophy; Nestin interacts with MAP1LC3B (LC3B), catalyzing its polyubiquitination to inhibit autophagy flux and suppress ferroptosis, whereas Nestin knockout exacerbates atrophy (Morgan et al., 2018b). Ferroptosis also occurs after skeletal muscle contusion, where iron overload activates the SAT1/ALOX15 pathway, increasing lipid peroxidation and impairing regeneration (Dubuisson et al., 2023). Moreover, exercise-induced protective effects against brain ischemia have been linked to skeletal muscle-derived exosomes carrying miR-484, which inhibit neuronal ferroptosis by targeting ACSL4, highlighting a novel muscle-brain crosstalk mediated by ferroptosis regulation (Kamiya et al., 2022). These findings collectively underscore ferroptosis as a central pathological mechanism in multiple muscle disorders and point to its potential as a therapeutic target. Therefore, targeting the regulation of ferroptosis via iron chelators in muscle satellite cells or myoblasts holds promise as a novel therapeutic strategy (Figure 2).

### 5.4 The role of pyroptosis in skeletal muscle

Inflammasome activation can stimulate dendritic cells (DC) to promote anti-tumor immunity and maintain immune surveillance functions (Zhivaki et al., 2020; Zhivaki and Kagan, 2021). However, excessive inflammasome activation is a key factor in muscle atrophy triggered by conditions such as sepsis (Liu et al., 2020; Dubuisson et al., 2021). In the context of sepsis, pathogen-associated molecular patterns (PAMPs; e.g., LPS) and damage-associated molecular patterns (DAMPs) are potent activators of the NLRP3 inflammasome in immune and muscle cells. The ensuing caspase-1 activation cleaves GSDMD to execute pyroptosis and processes pro-IL-1β/IL-18 into mature, highly inflammatory cytokines. *In vivo* and *in vitro* studies confirm that NLRP3 knockout reduces inflammation-induced skeletal muscle atrophy by lowering IL-1β expression (Huang et al., 2017). In the CLP mouse model, inhibiting the NLRP3/IL-1β pathway alleviates sepsis-induced cardiac muscle atrophy and cardiomyopathy (Busch et al., 2021). Furthermore, LPS-induced septic mice show significantly increased expression of NLRP3/IL-1β, MuRF1, and MAFbx. Yet, negative regulators of NLRP3, such as the double-stranded RNA-dependent PKR inhibitor, suppress these signals and improve muscle atrophy (Valentine et al., 2018). Knocking out NLRP3 or GSDMD also mitigates dexamethasone-induced pyroptosis and atrophy in myotubes. These findings collectively establish the NLRP3/GSDMD-mediated pyroptotic pathway as a central mechanistic driver of sepsis-induced muscle wasting (Figure 2). A recent study provides direct genetic evidence for this, demonstrating that GSDMD knockout alleviates sepsis-associated skeletal muscle atrophy in mice. This protective effect was achieved through inhibition of the IL18/AMPK signaling pathway, which subsequently suppressed the activation of both the ubiquitin-proteasome system and autophagy, key processes driving muscle protein degradation (Zhang Y. et al., 2024). The released IL-1β directly contributes to the proteolytic environment by upregulating ubiquitin-proteasome system components like MuRF1, linking inflammatory cell death directly to muscle protein degradation. This implies that targeting pyroptosis could be a viable strategy to preserve muscle mass in critically ill septic patients. Muscle atrophy in metabolic diseases is linked to pyroptosis as well. In type II diabetes, mitochondrial ROS accumulation activates the NLRP3 inflammasome (Wei and Cui, 2022), which then triggers the Caspase-1/GSDMD pathway and initiates pyroptosis in muscle cells. IL-1β released during pyroptosis further inhibits the insulin signaling pathway (Américo-Da-Silva et al., 2021; Jorquera et al., 2021), worsening insulin resistance and muscle atrophy. Thus, cumulative evidence from these studies suggests that the NLRP3 inflammasome plays a key role in mediating skeletal muscle atrophy under various pathological conditions.

Emerging research reveals that pyroptosis in muscle atrophy extends beyond the canonical NLRP3/Caspase-1/GSDMD axis. A study by Wu et al. identified a novel pathway in aged sarcopenic muscle, where the inflammatory cytokine TNF-α activates caspase-8 and caspase-3, which in turn cleave Gasdermin E (GSDME) to execute pyroptosis, leading directly to myotube loss (Wu et al., 2023). Conversely, interventional strategies targeting pyroptosis show therapeutic promise. Research by Narasimhulu and Singla (Narasimhulu and Singla, 2023) demonstrated that Bone Morphogenetic Protein-7 (BMP-7) alleviates diabetic myopathy by attenuating lipid accumulation and inflammation, which subsequently suppresses pyroptosis and mitigates sarcopenia and adverse muscle remodeling. These findings collectively underscore that targeting distinct pyroptotic pathways holds significant potential for developing tailored therapies against muscle wasting in both aging and metabolic diseases.

### 5.5 The role of autophagy in skeletal muscle

In skeletal muscle, the autophagy-lysosome pathway represents one of the most critical mechanisms regulating muscle metabolism. Autophagy plays an essential role in maintaining muscle mass and function. Exercise training induces autophagy in myocytes, which facilitates the clearance of damaged mitochondria and proteins, thereby enhancing muscle metabolism (Nassour et al., 2019; Vainshtein and Hood, 1985). However, both excessive activation and inhibition of autophagy can lead to muscle disorders, a phenomenon commonly observed in patients with sarcopenia (Yin et al., 2021; Herrenbruck and Bollinger, 2020). In aging muscle, a primary mechanistic defect is the decline in autophagic flux, leading to the accumulation of damaged organelles and proteins. Conversely, excessive autophagy can also be detrimental. For instance, oxidative stress triggered by muscle-specific expression of mutant superoxide dismutase (SOD1^G93A^) primarily induces muscle atrophy through autophagy activation. Knockdown of LC3 to reduce excessive autophagy has been shown to preserve muscle mass in SOD1^G93A^ transgenic mice, indicating that autophagy is activated during muscle breakdown (Dobrowolny et al., 2008; Sun et al., 2022; Yang et al., 2021). Initially regarded as a non-selective degradation pathway, autophagy is now understood to be triggered by specific stimuli such as protein aggregates, enabling the selective removal of organelles including mitochondria. Mitochondria are vital for muscle function and metabolism, undergoing dynamic changes in morphology and abundance in response to muscle activity. The mitochondrial network is remodeled through processes involving fusion and fission proteins, mitochondrial shaping mechanisms, and the selective elimination of small mitochondria via autophagy—a process termed mitophagy (Romanello and Sandri, 2013). Key regulators of mitophagy include Parkin, PINK1, and Bnip3; inactivation of their genes leads to mitochondrial abnormalities (Bothe et al., 2000). In atrophied muscle, the mitochondrial network undergoes substantial remodeling following fasting or denervation. Bnip3-mediated autophagy contributes significantly to these mitochondrial alterations (Mofarrahi et al., 2012). Furthermore, the upregulation of mitochondrial fission mechanisms induces muscle atrophy in mice, whereas inhibiting fission mitigates muscle loss during denervation (Romanello et al., 2010), underscoring that disruption of the mitochondrial network is a key mechanism in muscle atrophy. Supporting this, muscle-specific knockout mice lacking autophagy-related protein genes develop atrophy, weakness, and various myopathic features (Chang et al., 2012; Masiero et al., 2009). Furthermore, the protective role of basal autophagy is underscored in sepsis, a severe catabolic state. Contrary to what might be expected, muscle-specific ablation of the essential autophagy gene Atg7 in septic mice exacerbated muscle wasting, worsened whole-body metabolic derangements, and significantly decreased survival. This demonstrates that intact autophagic flux is a critical adaptive response and protective mechanism in septic skeletal muscle (Leduc-Gaudet et al., 2023b). Additionally, muscle biopsies from patients with congenital muscular dystrophy reveal reduced levels of Beclin-1 and Bnip3. Impaired mitophagy flux results in the accumulation of damaged and dysfunctional mitochondria, ultimately leading to myofiber degeneration (Grumati et al., 2010). Thus, autophagy serves as an important regulator of muscle atrophy: Impaired autophagic flux, whether insufficient or excessive, can disrupt intracellular homeostasis and contribute to muscle protein degradation. Given this, modulating autophagy presents a promising therapeutic strategy for muscle atrophy (Figure 2).

### 5.6 The role of other cell death in skeletal muscle

The primary form of cell death induced by copper overload is termed cuprotosis (Cao et al., 2023). Copper overload is closely associated with the development and progression of various skeletal muscle diseases (Chen et al., 2024). The copper transporter CTR1 which is responsible for copper uptake is widely expressed in skeletal muscle cells (Whitlow et al., 2023). During skeletal muscle ischemia-reperfusion injury CTR1 expression is upregulated and this promotes copper uptake. It leads to increased intracellular copper deposition and consequently exacerbates myoblast injury (Fu et al., 2024). Furthermore, cuproptosis has been proposed as a novel mechanism contributing to sepsis-acquired weakness by directly disrupting mitochondrial TCA cycle proteins, a process distinct from other known regulated cell death pathways (Kamiya et al., 2023). The clinical relevance is highlighted by the development of a diagnostic model for sarcopenia based on cuproptosis-related genes, which demonstrates high predictive accuracy and links this cell death process to age-related muscle decline (Guo et al., 2023). Parthanatos is a regulated cell death form that depends on poly (ADP-ribose) polymerase-1 (PARP-1). It causes cell death by inducing energy depletion and the release of apoptosis-inducing factor known as AIF (Wang et al., 2011; Yu et al., 2002). After myocardial infarction reperfusion reactive oxygen species and reactive nitrogen species are produced and they can activate PARP-1. This initiates the parthanatos process (Griendling et al., 2000). Studies in cardiomyocytes and mouse models suggest that the deubiquitinating enzyme MYSM1 regulates PARP1 activity and participates in parthanatos, thereby contributing to the development of cardiac hypertrophy (Zhong et al., 2025). Moreover, AIF mutations can disrupt mitochondrial respiratory chain function and enhance parthanatos-dependent cell death. This leads to severe mitochondrial encephalomyopathy (Ghezzi et al., 2010). Currently the role of parthanatos in cardiac diseases and encephalomyopathy is recognized but its specific role in skeletal muscle pathology remains to be further explored.

## 6 Targeting cell death pathways: therapeutic prospects for skeletal muscle disorders

The intricate involvement of various cell death pathways in skeletal muscle pathology presents a rich landscape for therapeutic intervention. Understanding these mechanisms has opened promising avenues for treating muscle diseases by specifically modulating apoptosis, necroptosis, pyroptosis, ferroptosis, and autophagy. The timely and effective clearance of death cells, a process highlighted as critical for muscle homeostasis and the prevention of autoimmunity, itself represents a therapeutic target to resolve inflammation and promote regeneration.

### 6.1 For apoptosis

While caspase inhibitors have long been explored as a therapeutic strategy, their systemic application remains challenging due to potential off-target effects and toxicity. Consequently, several natural products have demonstrated significant potential in mitigating skeletal muscle atrophy through multi-faceted actions on apoptotic and proteolytic pathways. For instance, curcumin has been shown to improve muscle recovery following immobilization by concurrently suppressing proteasome activity and caspase-9-mediated apoptosome activation, thereby creating a cellular environment conducive to regeneration (Vazeille et al., 2012). Similarly, resveratrol ameliorates muscle atrophy in diabetic models not only by reducing cleaved caspase-3 and ubiquitin ligase expression but also by comprehensively enhancing mitochondrial biogenesis and suppressing aberrant mitophagy—a process closely linked to apoptotic initiation (Wang et al., 2018). In aged muscle, apigenin exerts protective effects by alleviating oxidative stress and inhibiting hyperactive mitophagy and apoptosis, as reflected by reduced DNA fragmentation and BNIP3 levels, while concurrently promoting mitochondrial function (Wang D. et al., 2022). Furthermore, in denervation-induced atrophy, isoflavone supplementation significantly attenuates apoptosis and fiber atrophy, independent of atrogin-1-mediated proteolysis, underscoring its specific role in modulating cell death pathways (Tabata et al., 2019). Collectively, these studies highlight that natural compounds such as curcumin, resveratrol, apigenin, and isoflavones can effectively target both apoptotic and mitochondrial dysfunction pathways, offering promising translational strategies for treating diverse forms of muscle wasting.

### 6.2 For necroptosis

The RIPK1/RIPK3/MLKL axis is a prime therapeutic target for counteracting muscle pathology. Evidence across diverse disease models underscores the efficacy of targeting this pathway. In Duchenne muscular dystrophy (DMD), genetic ablation of Ripk3 in mdx mice significantly reduced myofibre degeneration, inflammation, and fibrosis, leading to improved muscle function, establishing RIPK3 as a key degenerative mediator (Morgan et al., 2018b). The adiponectin receptor agonist ALY688, have demonstrated efficacy in reducing muscle pathology in models like DMD (Dubuisson et al., 2023). Beyond genetic disorders, the role of necroptosis extends to inflammatory and systemic conditions. In inflammatory myopathies, muscle fibers specifically undergo necroptosis, which drives the release of damage-associated molecular patterns (DAMPs) like HMGB1, perpetuating inflammation and weakness. Strategies to break this cycle, such as pharmacological inhibition of necroptosis or neutralization of HMGB1, have been shown to ameliorate muscle cell death, inflammation, and functional deficits in experimental models (Kamiya et al., 2022; Kamiya et al., 2023). Furthermore, in a cachexia model induced by lipopolysaccharide, the small molecule necroptosis inhibitor Necrostatin-1 (targeting RIPK1) effectively alleviated muscle morphological damage, suppressed pro-inflammatory cytokine release, and attenuated the activation of the protein degradation signaling pathway (Guo et al., 2023). Collectively, these findings validate the RIPK1/RIPK3/MLKL axis as a central node in muscle wasting across etiologies and demonstrate the therapeutic potential of its inhibition.

### 6.3 For ferroptosis

GPX4 and system xc^−^ are central regulators of ferroptosis. Inhibitors of lipid peroxidation like ferrostatin-1, as well as antioxidants like CoQ10, have shown promise in preclinical models to protect myoblasts and preserve muscle mass (Xiang et al., 2025). The therapeutic potential of targeting this pathway is evident across various models of muscle atrophy. For instance, in aged skeletal muscle, iron accumulation activates a P53-SLC7A11 signaling axis, leading to lipid peroxidation and ferroptosis, which can be mitigated by the ferroptosis inhibitor ferrostatin-1 (Huang et al., 2021). Beyond direct inhibition, enhancing endogenous protective systems represents another strategic approach. The Cystathionine gamma-lyase (CSE)/Hydrogen Sulfide (H_2_S) system serves as a key endogenous guard against ferroptosis; its downregulation contributes to ferroptosis, while exogenous H_2_S can protect myoblasts by inhibiting the acetylation of ALOX12, a key initiator of membrane lipid peroxidation (Wang Y. et al., 2021). Furthermore, addressing the root cause of iron dyshomeostasis is critical. Research has shown that the age-related decline of Transferrin Receptor 1 (Tfr1) in muscle satellite cells leads to aberrant iron accumulation via the zinc transporter Zip14, triggering ferroptosis and severely impairing muscle regeneration. Importantly, intramuscular administration of Tfr1 via lentivirus was able to partially reverse this pathological process, highlighting Tfr1 gene therapy as a novel and mechanistic strategy for combating age-related muscle decline (Ding et al., 2021). Natural compounds also offer potential, as demonstrated by Lobetyolin, which has been shown to alleviate ferroptosis and muscle wasting in a chronic kidney disease model by activating the Hedgehog-GLI1 signaling pathway (116, Lobetyolin Alleviates Ferroptosis of Skeletal Muscle in 5/6 Nephrectomized Mice via Activation of Hedgehog-GLI1 Signaling). Collectively, these findings underscore a multi-pronged therapeutic approach against ferroptosis, encompassing iron chelation, enhancement of endogenous antioxidant systems (GPX4, H_2_S), and targeted gene therapy to restore iron homeostasis.

### 6.4 For pyroptosis

The NLRP3 inflammasome and its effector GSDMD are key druggable nodes in combating pyroptosis-driven muscle atrophy. Specific NLRP3 inhibitors, such as MCC950, have demonstrated remarkable efficacy in dystrophic muscle. In the mdx mouse model of DMD, MCC950 treatment significantly reduced inflammation, oxidative stress, myonecrosis, and fibrosis, leading to improved muscle maturation and function. This therapeutic benefit was mechanistically linked to the inhibition of the NLRP3-GSDMD axis, as evidenced by reduced levels of cleaved caspase-1 and N-terminal GSDMD, identifying pyroptosis as a previously unappreciated driver of pathology in DMD (Dubuisson et al., 2022). Beyond genetic disorders, targeting this pathway is also effective in metabolic and toxic models of atrophy. The anti-anginal drug Trimetazidine was shown to attenuate dexamethasone-induced muscle atrophy by suppressing NLRP3/GSDMD-mediated pyroptosis, an effect achieved through the restoration of the PI3K/AKT/FoxO3a signaling pathway (Wang L. et al., 2021). Similarly, in the context of diabetic nephropathy, the SGLT2 inhibitor Dapagliflozin alleviated skeletal muscle atrophy by directly binding to and suppressing GSDMD-mediated canonical pyroptosis (Zhang et al., 2025). Furthermore, therapeutic strategies that modulate upstream metabolic triggers of pyroptosis show great promise. In chronic kidney disease (CKD) models, the myokine Irisin ameliorated palmitic acid-induced muscle atrophy by inhibiting fatty acid oxidation and the subsequent NLRP3 inflammasome activation and pyroptosis (Zhou et al., 2023). Complementing this, Keto Acids supplementation was found to protect against CKD-induced muscle atrophy by concurrently inhibiting pyroptosis and upregulating the expression of FNDC5, the precursor of Irisin, establishing a powerful endogenous regulatory loop (Wang et al., 2025). These findings collectively underscore that therapeutic intervention at the level of the NLRP3 inflammasome, its effector GSDMD, or their upstream metabolic activators, constitutes a potent and versatile strategy for preserving muscle mass across a spectrum of diseases.

### 6.5 For autophagy

Modulating autophagy is a double-edged sword, requiring precise and context-dependent intervention. In conditions with impaired flux, such as aging, strategies to enhance autophagy can be highly beneficial. For instance, rejuvenating aged muscle can be achieved by inhibiting the prostaglandin-degrading enzyme 15-PGDH, which restores physiological levels of PGE2, leading to enhanced mitochondrial function and autophagy, and ultimately increasing muscle mass and strength (Palla et al., 2021). Similarly, directly activating the autophagy machinery has proven effective; muscle-specific overexpression of the autophagy regulator TP53INP2 was shown to prevent and even reverse sarcopenia in aged mice by enhancing autophagic activity and mitophagy (Sebastian et al., 2024). The therapeutic potential of boosting autophagy is further exemplified by the use of human umbilical cord mesenchymal stromal cells (hucMSCs) and their derived exosomes, which ameliorate diabetes- and obesity-induced muscle atrophy by activating the AMPK/ULK1 signaling pathway to enhance autophagic flux (Song et al., 2023). Conversely, in pathological states where autophagy is excessively activated, such as in diabetic muscle atrophy, targeted inhibition becomes therapeutically relevant. Zinc supplementation has been shown to alleviate diabetic muscle atrophy by acting through the GPR39 receptor to suppress the SIRT1/FoxO1 signaling axis, thereby curbing excessive autophagic degradation (Yu et al., 2025). Therefore, future strategies must move beyond generalized enhancement or suppression, aiming instead to restore physiological autophagic homeostasis in a disease- and context-specific manner.

Future success will likely depend on developing targeted delivery systems and rational combination therapies that address multiple pathways within the cell death network. Furthermore, the role of the immune system, particularly macrophages, in clearing dead cells and shaping the regenerative environment offers an additional layer for intervention (Song et al., 2023), aiming to modulate the tissue response to cell death rather than just preventing death itself.

## 7 Perspectives

Cell death exhibits complex regulatory networks and multidimensional functions in skeletal muscle physiology and pathology. Under physiological conditions programmed cell death and autophagy-dependent cell death clear damaged or senescent cells. This process maintains myonuclear domain homeostasis and coordinates satellite cell activation and differentiation thereby ensuring skeletal muscle regeneration capacity. However, under pathological conditions such as aging disuse atrophy and hereditary myopathies mitochondrial dysfunction oxidative stress and abnormal activation of inflammatory signaling pathways occur. These disruptions can lead to imbalances in apoptosis, necroptosis, pyroptosis, or ferroptosis. Consequently, this results in muscle atrophy regeneration failure and fibrosis deposition.

While our research had detailed each cell death pathway in isolation, it is crucial to recognize that they operate within an interconnected network in skeletal muscle physiology and pathology. Although the molecular executors and morphological features differ significantly among apoptosis, necroptosis, pyroptosis, ferroptosis, and autophagy, they often share common upstream triggers and engage in extensive, complex crosstalk, forming an integrated cell death network in skeletal muscle (Tang et al., 2019). Key shared stimuli include (Chen et al., 2023a): oxidative stress, where reactive oxygen species (ROS) can directly damage lipids to initiate ferroptosis (Liu et al., 2022), trigger mitochondrial outer membrane permeabilization to activate intrinsic apoptosis (Saelens et al., 2004), and serve as a critical activator for the NLRP3 inflammasome in pyroptosis (Wei and Cui, 2022); (Zhang et al., 2023) inflammatory cytokines, notably TNF-α, which can simultaneously engage pro-apoptotic signaling via caspase-8 and, upon caspase-8 inhibition, switch to activate the necroptotic machinery through RIPK1/RIPK3/MLKL (Holler et al., 2000; Cho et al., 2009); (Jiang et al., 2024) metabolic stress, such as nutrient deprivation or mitochondrial dysfunction, which can induce excessive autophagy, initiate intrinsic apoptosis via cytochrome c release (Chen et al., 2023b), and deplete key antioxidants like glutathione, thereby sensitizing cells to ferroptosis (Forman et al., 2009); and (Yamakawa et al., 2020) specific damage-associated molecular patterns (DAMPs) released from damaged muscle, which can activate pattern recognition receptors to instigate both pyroptosis and necroptosis (Tang et al., 2019; Broz and Dixit, 2016). Downstream, several effector mechanisms act as critical nodal points for pathway interplay. Mitochondrial dysfunction stands as a central hub, not only initiating the intrinsic apoptotic cascade, but also producing copious ROS that propagate lipid peroxidation in ferroptosis, and amplify inflammatory signaling for necroptosis and pyroptosis (He et al., 2011; Wang Y. et al., 2022). Caspase-8 functions as a decisive molecular switch at the crossroads of apoptosis and necroptosis; its activation leads to apoptotic cleavage of effector caspases, while its inhibition permits RIPK3-mediated phosphorylation of MLKL, committing the cell to necroptotic demise (Elmore, 2007; Wang et al., 2014). Furthermore, the interplay between inflammasome activation (pyroptosis) and the release of mature IL-1β/IL-18 creates a potent inflammatory microenvironment (Rathkey et al., 2018). These cytokines can, in turn, exacerbate muscle wasting by upregulating ubiquitin-proteasome components like MuRF1, thereby linking inflammatory cell death directly to protein degradation pathways and influencing the activity of other cell death modalities. The concept of PANoptosis, a coordinated cell death process involving apoptosis, necroptosis, and pyroptosis, has recently been demonstrated in muscle atrophy models, providing direct evidence for this functional crosstalk (Vazeille et al., 2012). This network view underscores that in skeletal muscle pathology, these pathways rarely operate in isolation. Consequently, therapeutic strategies may achieve greater efficacy by targeting shared upstream stressors (e.g., oxidative stress, inflammation) or critical nodal points (e.g., caspase-8, mitochondria) to restore cell death homeostasis, rather than focusing on a single pathway in isolation.

To translate this network understanding into tangible progress, future research should prioritize several concrete directions. First, there is a critical need to delineate the temporal sequence and hierarchy of cell death pathway activation. Longitudinal studies in disease models are required to determine whether different pathways are activated in a specific order (e.g., whether pyroptosis initiates inflammation that is subsequently amplified by ferroptosis and necroptosis) and to identify potential master regulators of this network. Second, the field must develop non-invasive, pathway-specific biomarkers. Discovering and validating circulating biomarkers (e.g., specific DAMPs, lipid peroxidation products, or microRNAs) for human use is essential for early diagnosis, patient stratification, and monitoring therapeutic responses. Third, future work must dissect the cell death crosstalk within specific cellular compartments of the muscle niche. Utilizing cell-type-specific knockout models and single-cell omics, researchers should investigate how these interactions differ between myofibers, satellite cells, and fibro-adipogenic progenitors (FAPs), as this will reveal cell-specific vulnerabilities and therapeutic targets. Finally, overcoming the challenge of therapeutic specificity is paramount. The development of innovative muscle-targeted delivery systems, such as adeno-associated viruses (AAVs) with muscle-specific promoters or advanced nanoparticle technologies, is crucial to minimize off-target effects when modulating these ubiquitous cell death pathways.

Understanding cell death pathways opens promising therapeutic avenues for muscle diseases. Key targets include: for ferroptosis, GPX4 and system xc^−^ (e.g., SLC7A11), with inhibitors like ferrostatin-1 and CoQ10 showing promise in preclinical models (Xiang et al., 2025); for necroptosis, the RIPK1/RIPK3/MLKL axis, targeted by agents like the adiponectin receptor agonist ALY688 (Dubuisson et al., 2023); and for pyroptosis, the NLRP3 inflammasome and GSDMD. Natural compounds, such as Lobetyolin, also demonstrate potential by modulating these pathways (Wang Y. et al., 2021). However, clinical translation faces significant hurdles. The ubiquitous nature of cell death mechanisms raises concerns about systemic toxicity. Furthermore, pathway crosstalk and redundancy may lead to compensatory activation when one pathway is inhibited. Finally, achieving muscle-specific drug delivery remains a major challenge. Future success will likely depend on developing targeted delivery systems and rational combination therapies.

The mechanisms of cell death in skeletal muscle are complex and not fully elucidated. As a highly plastic tissue research on skeletal muscle cell death mechanisms not only provides therapeutic targets for degenerative diseases but also reveals deep principles of cell fate in regenerative medicine. In summary future studies should further investigate the molecular mechanisms and develop corresponding therapeutic strategies to improve treatment outcomes for skeletal muscle-related diseases and enhance patients’ quality of life.
